# Xiao-Qing-Long Tang Prevents Cardiomyocyte Hypertrophy, Fibrosis, and the Development of Heart Failure with Preserved Ejection Faction in Rats by Modulating the Composition of the Gut Microbiota

**DOI:** 10.1155/2019/9637479

**Published:** 2019-07-18

**Authors:** Guo-Feng Zhou, Yue-Hua Jiang, Du-Fang Ma, Yong-Cheng Wang, Jin-Long Yang, Ji-Ye Chen, Chen-Yu Chi, Xiao-wei Han, Zhao-Yu Li, Xiao Li

**Affiliations:** ^1^Shandong University of Traditional Chinese Medicine, Jinan, Shandong 250000, China; ^2^Affiliated Hospital of Shandong University of Traditional Chinese Medicine, Jinan, Shandong 250011, China

## Abstract

**Background:**

Changes in the gut microbiota are associated with cardiovascular disease progression. Xiao-Qing-Long Tang (XQLT), a traditional herbal formula, has an anti-inflammatory effect and regulates the steady state of the immune system, which is also associated with the progression of heart failure with preserved ejection faction (HFpEF). In this study, we investigated whether XQLT could contribute to prevent the development of HFpEF and whether the modulation of the gut microbiota by this herbal formula could be involved in such effect.

**Methods:**

The gut microbiota, SCFAs, the histology/function of the heart, and systolic blood pressure were examined to evaluate the effect of XQLT on the gut microbiota and the progression of HFpEF after oral administration of XQLT to model rats. Furthermore, we evaluated, through fecal microbiota transplantation experiments, whether the favorable effects of XQLT could be mediated by the gut microbiota.

**Results:**

Oral administration of XQLT contributed to the reduction of elevated blood pressure, inflammation, and compensatory hypertrophy, features that are associated with the progression of HFpEF. The gut microbiota composition, SCFA levels, and intestinal mucosal histology were improved after treatment with XQLT. Moreover, fecal transfer from XQLT-treated rats was sufficient to prevent the progression of HFpEF.

**Conclusions:**

These data suggested that XQLT prevented the development of HFpEF in model rats by regulating the composition of the gut microbiota.

## 1. Introduction

Heart failure with preserved ejection faction (HFpEF) is a common type of heart failure with poor outcomes which accounts for more than 50% of cases of heart failure (HF) [1, 2]. Although advances have been made in the pathophysiological characterization and treatment of HFpEF [3], there is still lack of effective drugs [4]. In addition, HFpEF prevalence has been increasing with metabolic disease epidemics and the increasing age of the population [5]. HFpEF remains a public health problem. Inflammatory activation leads to microvascular ischemia and left ventricular fibrosis [6] and promotes the progression of HFpEF [7–12]. In addition, recent studies have shown that anti-inflammatory strategies have achieved promising results in animal models of HFpEF [6]. This evidence indicates that chronic inflammation plays an important role in HFpEF.

Recent evidence indicates that gut dysbiosis is associated with the development of cardiovascular disease and its associated chronic inflammation [13, 14]. In addition, some studies have shown that gut dysbiosis impairs intestinal integrity, leading to the leakage of harmful metabolites from specific bacteria into the bloodstream and contributing to the development of chronic inflammation [15, 16]. Therefore, regulating the gut microbiota may contribute to ameliorating the progression of HFpEF.

The traditional Chinese medicinal compound Xiao-Qing-Long Tang (XQLT), also known as Sho-Seiryu-To in Japan, exhibits anti-inflammatory and antifibrotic effects in the lung tissues of mice exposed to cigarette smoke concentrate (CSC) and lipopolysaccharide (LPS) [17]. In fact, some molecular pathways and characteristics of inflammation are simultaneously present in myocardial tissue and lung tissue. Furthermore, recent evidence has indicated that XQLT influences gastrointestinal motility [18]. This evidence suggests that XQLT may prevent the progression of HFpEF by regulating the composition of the gut microbiota. Therefore, we tried to investigate whether XQLT could contribute to preventing the development of HFpEF and whether the modulation of the gut microbiota by XQLT could be involved in such effect.

Our study suggested that XQLT administration conduced to beneficial effects on the progression of HFpEF, lowering systolic blood pressure and exhibiting anti-inflammatory and antifibrotic effects. Supplementation with XQLT contributed to changes in the composition of the gut microbiota and increased the levels of acetate and butyrate, changes that were associated with prevention of the progression of HFpEF. Furthermore, fecal transfer from XQLT-treated rats also contributed to similar favorable effects. The data suggested that XQLT prevented the progression of HFpEF by regulating the composition of the gut microbiota in model rats.

## 2. Methods and Materials

All animal experiments complied with international guidelines for the care and use of laboratory animals. The animal protocols were reviewed and approved by the Institutional Animal Care and Use Committee of Shandong University of Traditional Chinese Medicine.

### 2.1. Animals and Experimental Protocols

**Figure 1:**
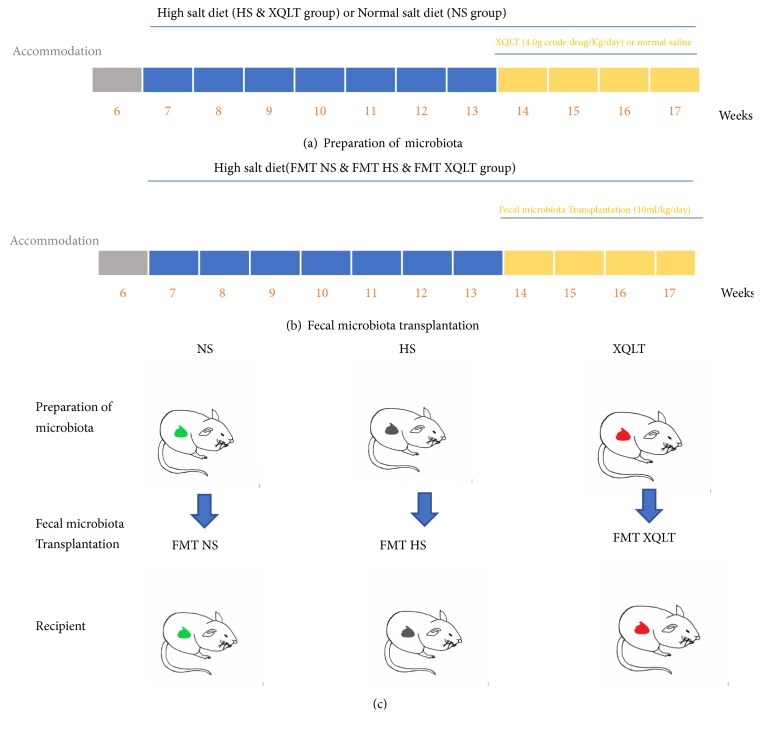
Study design. The preparation of the microbiota (a) is described in Sections 3.1–3.6 and in the second paragraph of the Discussion. Fecal microbiota transplantation (b) is described in Sections 3.7to 3.8 and in the third paragraph of the Discussion. The prepared gut microbiota was transplanted into the corresponding groups (c) as described in the Materials and Methods.

Six-week-old specific pathogen-free male Dahl salt-sensitive rats weighing 160–180 g (Certificate No. 2016-0006) were obtained from the Charles River Animal Laboratory (Beijing, China). The rats were housed in controlled conditions (temperature: 20±2°C; light: 12 h light-dark cycle; relative humidity: 40–60%) for one week to accommodate to the environment and were randomly divided into three groups (Figure 1(a)): the NS group, which was fed an NSD (0.3% NaCl chow and oral administration of normal saline at 2 ml/day from 14 weeks of age, n=6) for 11 weeks; the HS group, which was fed an HSD (8% NaCl chow and oral administration of normal saline at 2 ml/day from 14 weeks of age, n=6) for 11 weeks; and the XQLT group, which was fed an HSD and treated with XQLT (8% NaCl chow and oral administration of XQLT at 2 ml/4.0 g crude drug/kg/day from 14 weeks of age, n=6). The rats were housed individually to prevent a cohousing effect on the microbiota. Systolic blood pressure measurement was performed with a 12-channel tail-cuff blood pressure system (MRBP, IITC Life Science Instruments, USA) every week after the adaptation week.

### 2.2. Preparation of the XQLT Decoction

The XQLT formula was composed of eight medicinal herbs at a ratio of 3:3:1:2:1:2:1:3: Ma-Huang (Herba Ephedrae), Bai-Shao (Radix Paeoniae Alba), Xi-Xin (Herba Asari), Gan- Cao (Radix Glycyrrhizae), Gan-Jiang (Rhizoma Zingiberis), Gui-Zhi (Ramulus Cinnamomi), Wu-Wei-Zi (Fructus Schisandrae Chinensis), and Ban-Xia (Rhizoma Pinelliae). All of the herbs were provided by the Affiliated Hospital of Shandong University of Traditional Chinese Medicine (Jinan, China) and were identified by Prof. Feng Li. The XQLT formula was extracted under reflux with distilled water (1:10 volumes) twice for 1 h each. The extracts were mixed, filtered, and concentrated to a relative density of 1.20–1.25 (70–80°C). Excipient granules of powdered erythritol and stevioside (99:1) were added, and the mixture was dried below 60°C to obtain XQLT granules. Two grams of initial drug were contained in 1 gram of granules. An aqueous solution with 2 g crude drug/mL was used for the experiments.

### 2.3. Fecal Microbiota Transplantation

Fecal samples were collected and pooled from the rats of the NS, HS, and XQLT groups (at 18 weeks of age) and stored at -80°C. Fifteen specific pathogen-free male Dahl salt-sensitive rats (six weeks old, 160-200 g, Charles River Animal Laboratory) were randomly assigned into three groups after the adaption week (Figures 1(b) and 1(c)): the FMT NS group (8% NaCl chow, inoculated daily with the gut flora from rats of the NS group for the final 4 weeks, n=5), the FMT HS group (8% NaCl chow, inoculated daily with the gut flora from rats of the HS group for the final 4 weeks, n=5), and the FMT XQLT group (8% NaCl chow, inoculated daily with the gut flora from XQLT-treated rats for the final 4 weeks, n=5). The fecal samples were diluted 1:10 in sterile 0.9% NaCl, vortexed for 3 min, and centrifuged at 3000 rpm for 3 min. The recipient rats were administered the prepared fecal samples by gavage (10 ml/kg).

### 2.4. Echocardiographic Study

Echocardiographic studies were performed during the adaption week (when the rats were 6 weeks old), before treatment (when the rats were 14 weeks old) and after treatment (when the rats were 18 weeks old). The rats were placed under general anesthesia with sodium pentobarbital (20 mg/kg, i.p.). The parasternal short-axis and long-axis views were used to measure the function and mass of the left ventricle (LV) with an M5 Vet Veterinary Ultrasound system (Mindray, Guangdong, China). The M-mode measurements were used to assess the left ventricular mass (LV mass), left ventricular wall thickness (WT), left ventricular diastolic diameter (LVDd), and left ventricular ejection fraction (LVEF).

### 2.5. Tissue and Plasma Collection

After treatment, the rats were anaesthetized with sodium pentobarbital (20 mg/kg, intraperitoneal injection). Blood was collected from the main abdominal vein into a plasma separator tube, and the samples were centrifuged at 1000 x* g* for 15 min at 4°C. The samples were stored at -80°C until use. The LV and small intestine were collected from each rat, and the samples were divided into two subsets. One subset was fixed in 6% formaldehyde for hematoxylin-eosin (HE) and Masson's trichrome (Masson) staining, and another subset was stored at -80°C for western blot analysis and quantitative real-time PCR (qRT-PCR).

### 2.6. Histology Studies

The left ventricles and small intestines that were fixed with 6% formaldehyde and cut with a microtome were stained with Masson (for pathological analysis) and HE (for analysis of fibrotic areas). Hematoxylin-eosin staining was used to measure the numbers of infiltrating cells (the average numbers of macrophages and lymphocytes per 2 mm in 5 fields).

### 2.7. Analysis of the Gut Microbiota

Fecal samples were collected from the rats immediately after defecation after treatment and stored at -80°C. The hexadecyltrimethylammonium bromide (CTAB) method of Doyle and Doyle (1987), with modifications as described by Griffith and Shaw (1998), was used to extract total genomic DNA from the samples [19]. CTAB was purchased from Tianjin Guangfu Fine Chemical Research Institute. The concentration and purity of the DNA were monitored with 1% agarose gels, and the DNA was diluted to 1 ng/L. Specific primers (515F and 806R) were used to sequence the V4 region of bacterial 16S rRNA. An Ion Plus Fragment Library Kit 48 rxns (Thermo Scientific) was used to sequence the libraries, which were qualified with a Qubit® 2.0 Fluorometer (Thermo Scientific). The data were processed with Cutadapt [20] and compared with the SILVA database [21]. UPARSE software was used to analyze the sequences [22]. R software and the WGCNA package were used to conduct principal component analysis (PCA) for clustering, principal coordinate analysis (PCoA) for visualization of the data, and nonmetric multidimensional scaling (NMDS) for clustering of different samples, and the linear discriminant analysis effect size (LEfSe) was used to select OTUs that exhibited significance in structural segregation among the grouping of samples.

### 2.8. Fecal Short-Chain Fatty Acid Quantification

Fecal samples (0.2 g) were dissolved in ultrapure water (1 ml) and centrifuged at 12000 ×* g* for 20 min at 4°C. The supernatants were taken after centrifugation for GC analysis. The mixing standard was prepared with acetate (Sigma, 151785), butyrate (Sigma, B5887), and propionate (Sigma, 96727), after which GC analysis was performed. The GC system was equipped with an FID and an HP-FFAP column (30 m×0.32 mm×0.25 *μ*m, Agilent), and the carrier gas was helium. The GC conditions were as follows: the initial temperature of the HP-FFAP column was 60°C; then, the temperature increased to 150°C at a rate of 8°C/min, was maintained for 1 min, and increased to 220°C at a rate of 40°C/min for 2 min. The temperature of the injector and the FID was 250°C. The injection volume was 0.5 *μ*l, and the gas was a mixture of hydrogen, air, and nitrogen at a ratio of 30:300:20 mL/min.

### 2.9. Western Blot Analysis

Western blot analysis is an efficient method to quantify the protein levels of cytokines. The main pathological feature of fibrosis is the deposition of large amounts of extracellular matrix. During the fibrosis process, collagen I in the extracellular matrix gradually replaces collagen III, promoting the progression of fibrosis. We quantified the protein levels of collagen I and collagen III to evaluate the progression of fibrosis. In myocardial tissue, inflammatory factors (MCP-1, TNF-*α*, and IL-6) act by binding to related receptors. The protein levels of inflammatory factors can reflect the severity of inflammation. The left ventricular tissue samples were lysed in ice-cold phenylmethylsulfonyl fluoride (PMSF, Cat. No. ST506, Beyotime Biotechnology) and radioimmunoprecipitation assay (RIPA) buffer (Cat. No. P0013B, Beyotime Biotechnology). A bicinchoninic acid (BCA) assay (Cat. No. P0010, Beyotime Biotechnology) was used to measure the levels of total protein. The protein samples were denatured (95°C, 10 min) and electrophoretically separated by 8%-12% SDS-PAGE (Cat. No. P0012A, Beyotime Biotechnology), followed by transfer to PVDF membranes. The PVDF membranes were incubated with collagen I (Cat. No. ab34710, Abcam, 1:1000), collagen III (Cat. No. ab6310, Abcam, 1:1000), MCP-1 (Cat. No. ab25124, Abcam, 1:2000), TNF-*α* (Cat. No. ab6671, Abcam, 1:200), IL-6 (Cat. No. ab193753, Abcam, 1:1000), and actin (Cat. No. ab8226, Abcam, 1:5000) antibodies (with actin as the control) before being incubated with secondary antibodies. The protein bands were visualized by enhanced chemiluminescence and quantified with FluorChem Q 3.4 system (ProteinSimple, USA).

### 2.10. Statistical Analysis

The data were analyzed with SPSS 22.0 software (SPSS, USA) and are shown as the mean±standard deviation. To compare means among multiple groups, the data were analyzed by one-way analysis of variance (ANOVA), followed by Student's t-test. P<0.05 was considered to indicate statistical significance.

## 3. Results

### 3.1. XQLT Regulated Systolic Blood Pressure and Body Weight

**Figure 2:**
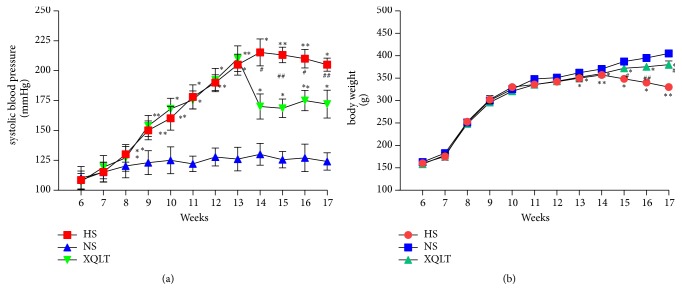
XQLT reduces systolic blood pressure and increases body weight in rats with heart failure with preserved ejection fraction (HFpEF). The effects of XQLT treatment on (a) systolic blood pressure and (b) body weight are shown. The data was presented as the mean±SD. (n=6 rats per group) *∗* p < 0.05 and *∗∗* p < 0.01 compared with the NS group; # p <0.05 and ## p <0.01 compared with the HS group.

Systolic blood pressure in all hypertensive groups was significantly increased (p= 0.38) and clearly higher than that in the NS group (NS:126±9.94 mmHg, HS: 205±8.79 mmHg, XQLT: 210±10.8 mmHg at 13 weeks; p<0.01, Figure 2(a)). Treatment with XQLT contributed to a reduction in blood pressure (NS: 124±7.28 mmHg, HS: 205±5.61 mmHg, XQLT: 172±11.7 mmHg at 17 weeks; p<0.05 Figure 2(a)). The body weight of the rats in all groups showed an increasing trend throughout the experiment. However, the increase in the HS group was significantly smaller than that in the NS group throughout the experiment (NS: 405±4.96 g, HS: 330±7.62 g, XQLT: 380±8.5 g at 17 weeks, p<0.05 Figure 2(b)).

### 3.2. XQLT Contributed to an Improvement in Cardiac Function and Ameliorated Changes in Organ Weight

**Figure 3:**
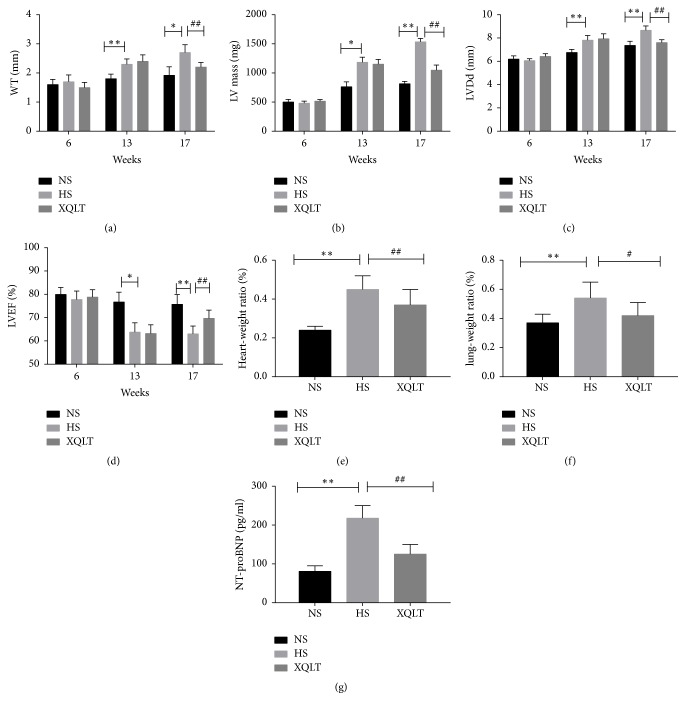
XQLT improves echocardiographic indexes, protects cardiac function, and ameliorates organ weight changes. Echocardiographic indexes such as the (a) wall thickness (WT), (b) left ventricular mass (LV mass), (c) left ventricular diastolic diameter (LVDd), and (d) left ventricular ejection fraction (LVEF) were assessed using an M5 Vet Veterinary Ultrasound system. The (e) heart weight/body weight and (f) lung weight/body weight ratios are shown. (g) NT-proBNP was assessed by ELISA. The data are presented as the mean±SD (n=6 rats per group) *∗* p < 0.05 and *∗∗* p < 0.01 compared with the NS group; # p <0.05 and ## p <0.01 compared with the HS group.

The left ventricular wall thickness (WT) and left ventricular mass (LV mass) were higher in the HS group than in the NS group, but treatment with XQLT conduced to attenuate the increase (WT, NS: 1.92±0.29 mm, HS: 2.7±0.27 mm, XQLT: 2.2±0.16 mm at 17 weeks; p<0.01, Figure 3(a)) (LV mass, NS: 814±41.59 mg, HS: 1534±59.41 mg, XQLT: 1048±88.43 mg at 17 weeks; p<0.01, Figure 3(b)). In the XQLT group, the increase in left ventricular diastolic diameter (LVDd) was significantly attenuated compared with that in the HS group (LVDd, NS: 7.36±0.36 mm, HS: 8.66±0.38 mm, XQLT: 7.6±0.25 mm at 17 weeks; p<0.01, Figure 3(c)). Supplementation with XQLT contributed to an increase in left ventricular ejection fraction (LVEF); however, the XQLT group had a lower LVEF than the NS group at 17 weeks (NS: 75.67±4.23%, HS: 63±3.41%, XQLT: 69.67±3.56%; p<0.01, Figure 3(d)). NT-proBNP levels were used to measure heart function. NT-proBNP levels in the HS group were significantly increased compared with those in the NS group, and supplementation with XQLT had a favorable effect on NT-proBNP (p<0.01, Figure 3(g)). The heart weight ratio was significantly increased in all hypertensive groups and was clearly higher in the hypertensive groups than in the NS group. However, the heart weight ratio of the XQLT group was significantly smaller than that of the HS group (NS: 0.24±0.02, HS: 0.45±0.07, XQLT: 0.37±0.08, p<0.01, Figure 3(e)). The lung weight ratio of the HS group was clearly higher than that of the NS group (p<0.01), but treatment with XQLT contributed to a significant reduction (NS: 0.37±0.06, HS: 0.54±0.11, XQLT: 0.42±0.09, p<0.05, Figure 3(f)).

### 3.3. Antifibrotic Effect of XQLT

**Figure 4:**
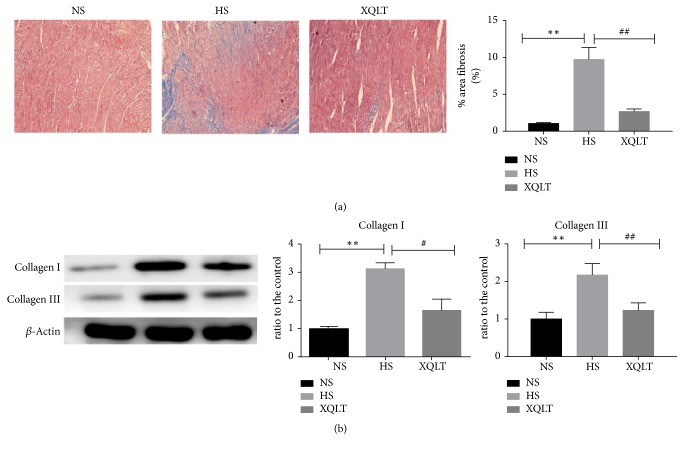
Treatment with XQLT prevents fibrosis in rats with heart failure with preserved ejection fraction (HFpEF). (a) Masson staining of the left ventricle is shown; the % area of fibrosis was assessed by analysis of the Masson staining. (b) The fibrosis-associated protein expression of collagen I and collagen III in the left ventricle was assessed using western blot analysis and was compared with that in the NS group. The data are presented as the mean±SD (n=6 rats per group). *∗* p < 0.05 and *∗∗* p < 0.01 compared with the NS group; # p <0.05 and ## p <0.01 compared with the HS group.

Masson staining showed that there was significantly less subendocardial fibrosis of the left ventricle in the XQLT group than in the HS group, although the subendocardial fibrosis of the LV in the XQLT group was higher than that in the NS group (NS: 1.02±0.19%, HS: 9±1.58%, XQLT: 2.46±0.32%, p<0.01, Figure 4(a)). Furthermore, the XQLT group showed lower levels of collagen I and collagen III (quantified by western blotting) than the HS group (Figure 4(b)).

### 3.4. Anti-Inflammatory Effect of XQLT

**Figure 5:**
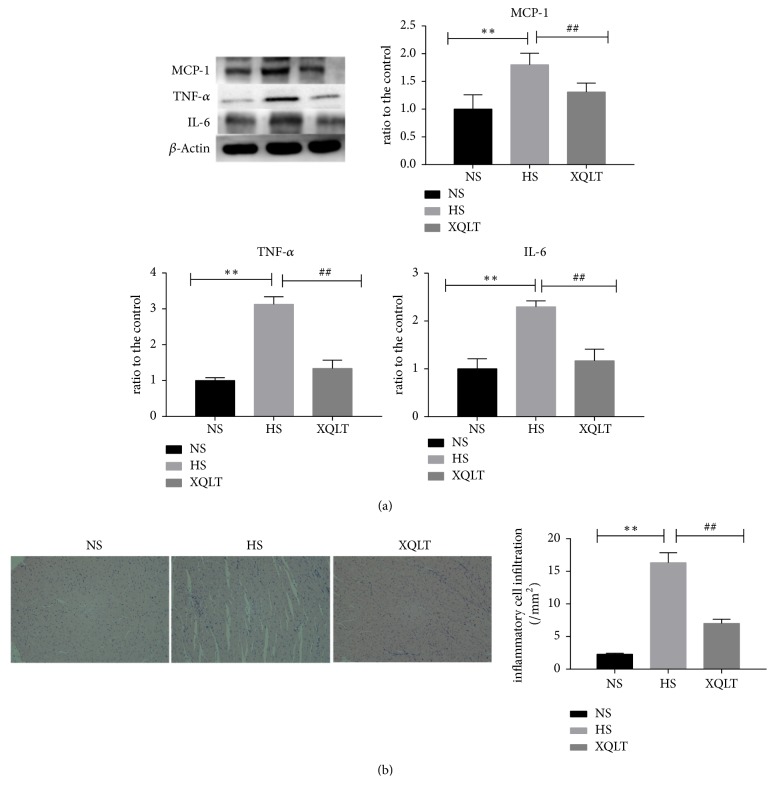
Treatment with XQLT prevented inflammation in rats with heart failure with preserved ejection fraction (HFpEF). (a) The inflammation-associated protein expression of MCP-1, TNF-*α*, and IL-6 in the left ventricle was assessed using western blot analysis and was compared with that in the NS group. (b) HE staining of the left ventricle is shown; inflammatory cell infiltration was assessed by analysis of the HE staining. The data are presented as the mean±SD (n=6 rats per group). *∗* p < 0.05 and *∗∗* p < 0.01 compared with the NS group; # p <0.05 and ## p <0.01 compared with the HS group.

The levels of MCP-1, TNF-*α*, and IL-6 (quantified by western blotting) in left ventricular tissue were significantly increased in the HS group compared with the NS group but were all decreased after treatment with XQLT (Figure 5(a)). Concomitantly, HE staining showed that the increase in inflammatory cell infiltration was significantly reduced after treatment with XQLT (Figure 5(b)).

### 3.5. XQLT Regulated the Histology and Tight Junctions of the Small Intestine and Ameliorates Endotoxemia

**Figure 6:**
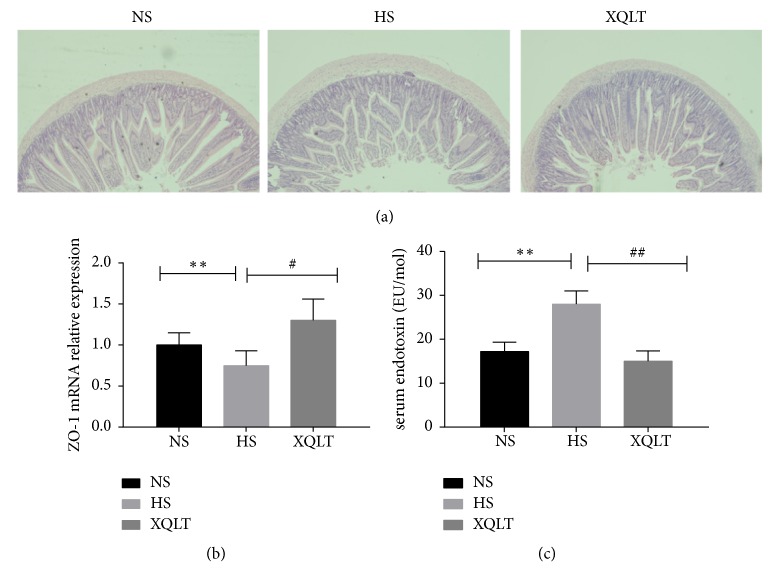
The beneficial effects of XQLT on the small intestine and on endotoxemia. (a) HE staining of the small intestine is shown. (b) ZO-1 mRNA expression in the intestinal mucosa was assessed using qRT-PCR and was compared with that in the NS group. (c) The levels of serum endotoxin are shown. The data are presented as the mean±SD (n=6 rats per group). *∗* p < 0.05 and *∗∗* p < 0.01 compared with the NS group; # p <0.05 and ## p <0.01 compared with the HS group.

Then HE staining showed that intestinal mucosal injury was significantly milder in the XQLT group than in the HS group, although intestinal mucosal injury was more serious in the XQLT group than in the NS group (Figure 6(a)). Furthermore, qRT-PCR showed that ZO-1 mRNA expression in the small intestine was significantly increased after administration of XQLT, although the XQLT group showed higher levels of ZO-1 than the NS group (p<0.01, Figure 6(b)). In addition, the level of serum endotoxin was significantly increased in the hypertension groups and decreased in the XQLT group compared to the HS group (p<0.01, Figure 6(c)).

### 3.6. XQLT Contributed to Reversal of Gut Dysbiosis and Increased Acetate and Butyrate Concentrations

**Figure 7:**
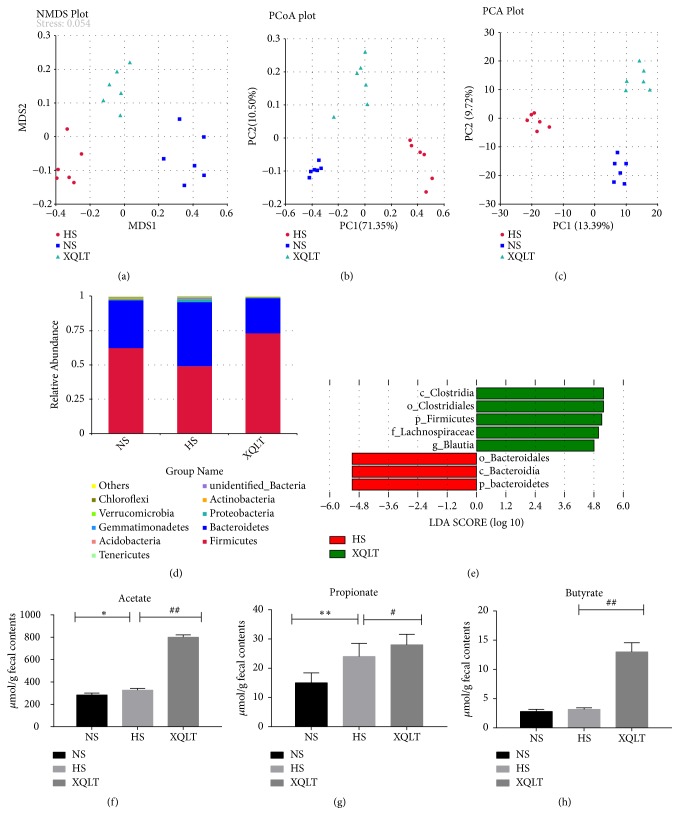
Effect of XQLT on the gut microbiota. (a) Nonmetric multidimensional scaling (NMDS), (b) principal component analysis (PCA), and (c) principal coordinate analysis (PCoA) were performed between the NS group, the HS group, and the XQLT group. (d) Composition of the bacteria at the genus level. (e) LEfSe analysis of the bacterial taxa between the HS group and the XQLT group. (f, g, h) The levels of (f) acetate, (g) propionate, and (h) butyrate in feces. The data are presented as the mean± SD (n=6 rats per group). *∗* p < 0.05 and *∗∗* p < 0.01 compared with the NS group; # p <0.05 and ## p <0.01 compared with the HS group.

We investigated whether XQLT supplementation influenced the structure of the gut microbiota by sequencing bacterial 16S rRNA in rats at 11 weeks. Weighted PCoA, PCA, and NMDS analyses were used to provide an overview of the structure of the gut microbiota in all groups at 11 weeks (Figures 7(a), 7(b), and 7(c)). The gut microbiota compositions of the NS group, HS group, and XQLT group at 11 weeks were significantly separated, indicating that administration of XQLT contributed to changes in the composition of the gut microbiota. At the phylum level, the ratio between Firmicutes and Bacteroidetes was used to evaluate gut dysbiosis. The ratio was significantly increased in the HS group compared with the NS group, but the XQLT group exhibited a lower ratio than the HS group (Figure 7(d)). At the genus level, the XQLT-treated group exhibited lower abundance of Proteobacteria and Bacteroidetes than the HS group (Figure 7(d)). LEfSe analysis showed that the beneficial effect of XQLT may be associated with changes at the genus level (Figure 7(e)). All hypertensive groups had higher levels of acetate and propionate than the NS group, and XQLT supplementation had a favorable effect on acetate levels (Figures 7(f) and 7(g)). The concentration of butyrate was not significantly different between the HS group and the NS group but was markedly increased in the XQLT group (Figure 7(h)).

### 3.7. XQLT Fecal Transposition Delayed the Progression of HFpEF

**Figure 8:**
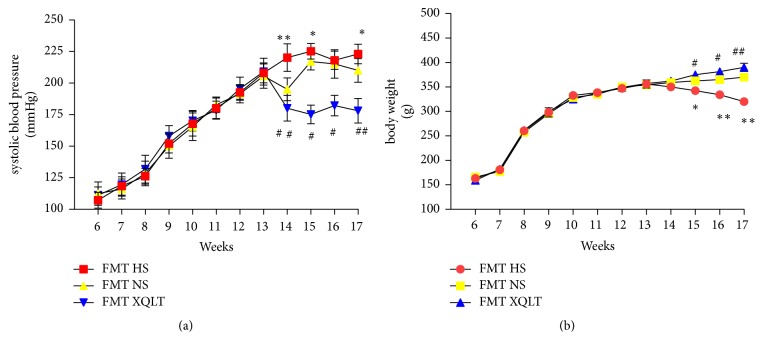
Effects of fecal transplantation from XQLT-treated rats on systolic blood pressure and body weight. Compared to that from rats in the FMT HS group, fecal transfer from rats in the NS group or the XQLT group reduced the increase in (a) systolic blood pressure and delayed the loss of (b) body weight. The data are presented as the mean± SD (n=5 rats per group). *∗* p < 0.05 and *∗∗* p < 0.01 compared with the FMT NS group; # p <0.05 and ## p <0.01 compared with the FMT HS group.

**Figure 9:**
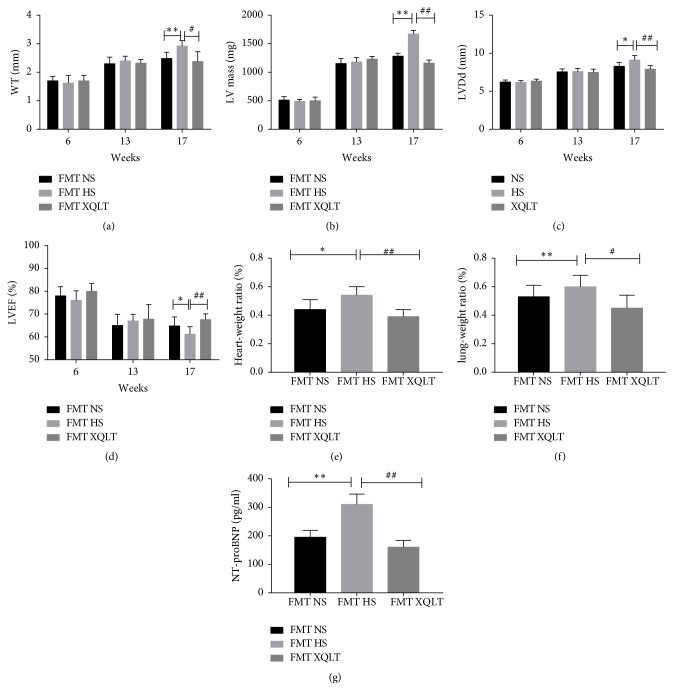
Effects of fecal transplantation from XQLT-treated rats on echocardiography measurements, organ weights, and blood test results. Seven-week-old specific pathogen-free male Dahl salt-sensitive rats were fed 8% NaCl chow for 11 weeks and colonized with fecal samples from different groups for 4 weeks. Then, the (a) wall thickness (WT) (b) left ventricular mass (LV mass), (c) left ventricular diastolic diameter (LVDd), (d) left ventricular ejection fraction (LVEF), (e) heart weight/body weight ratios, (f) lung weight/body weight ratios, and (g) NT-proBNP levels were measured. The data are presented as the mean± SD (n=5 rats per group). *∗* p < 0.05 and *∗∗* p < 0.01 compared with the FMT NS group; # p <0.05 and ## p <0.01 compared with the FMT HS group.

**Figure 10:**
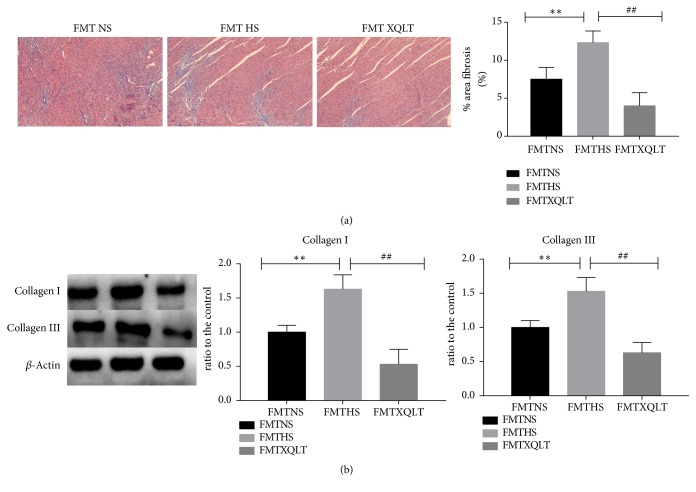
Analysis of fibrosis following transplantation from XQLT-treated rats. (a) Masson staining of the left ventricle and the area of fibrosis. (b) The fibrosis-associated protein expression of collagen I and collagen III in the left ventricle was assessed using western blot analysis and was compared with that in the FMT NS group. The data are presented as the mean± SD (n=5 rats per group). *∗* p < 0.05 and *∗∗* p < 0.01 compared with the FMT NS group; # p <0.05 and ## p <0.01 compared with the FMT HS group.

**Figure 11:**
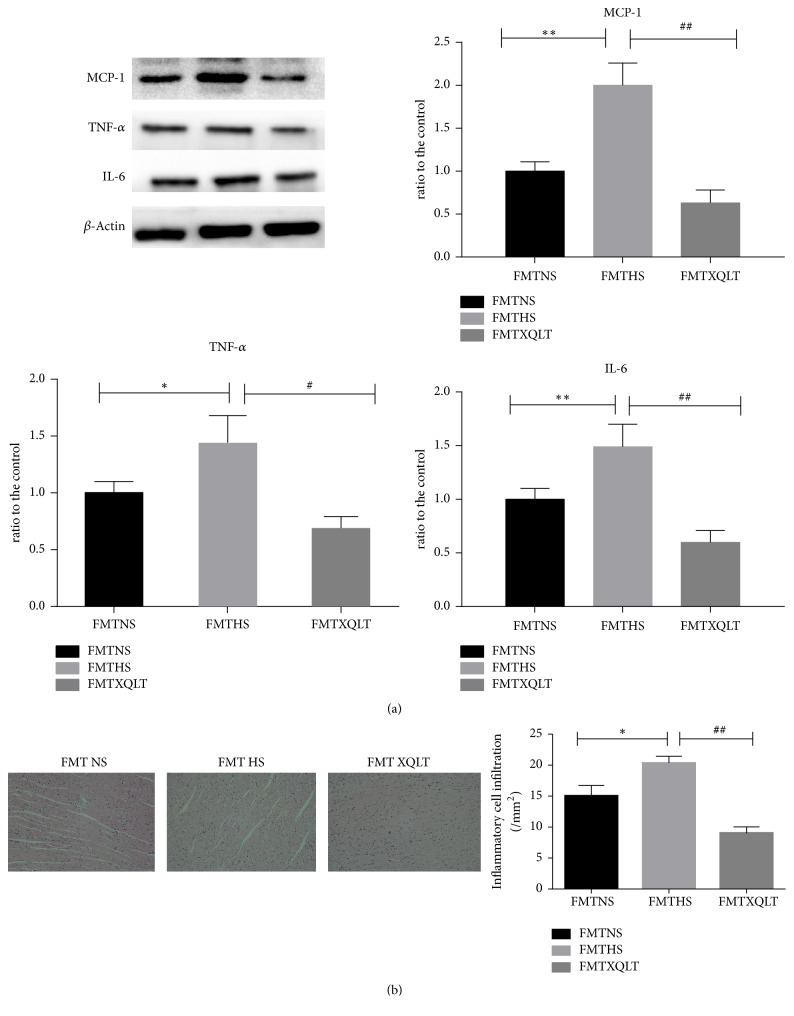
Effects of fecal transposition from XQLT-treated rats on proinflammatory cytokines and inflammatory cell infiltration. (a) The inflammation-associated protein expression of MCP-1, TNF-*α*, and IL-6 in the left ventricle was assessed using western blot analysis and was compared with that in the FMT NS group. (b) HE staining of the left ventricle is shown; inflammatory cell infiltration was assessed by analysis of the HE staining. The data are presented as the mean± SD (n=5 rats per group). *∗* p < 0.05 and *∗∗* p < 0.01 compared with the FMT NS group; # p <0.05 and ## p <0.01 compared with the FMT HS group.

**Figure 12:**
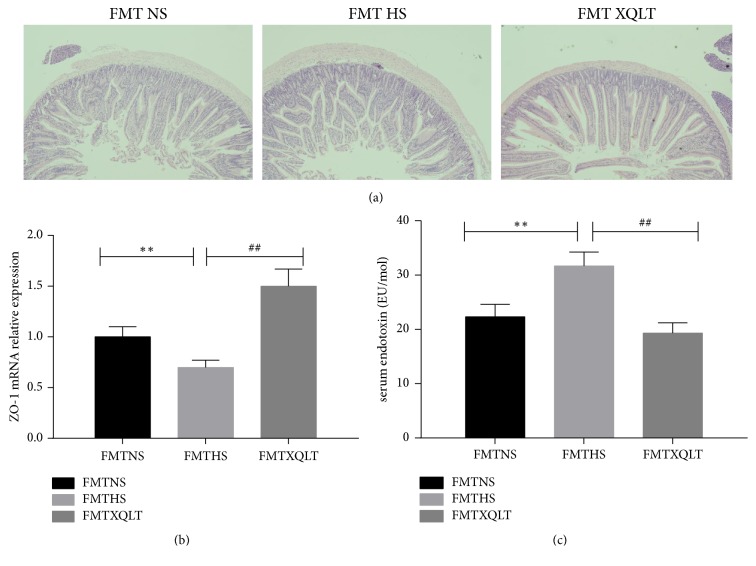
Effect of fecal transposition from XQLT-treated rats on the small intestine. (a) HE staining of the small intestine. (b) The expression of ZO-1 in the intestinal mucosa was assessed using qRT-PCR and was compared with that in the FMT NS group. (c) The levels of serum endotoxin. The data are presented as the mean± SD (n=5 rats per group). *∗* p < 0.05 and *∗∗* p < 0.01 compared with the FMT NS group; # p <0.05 and ## p <0.01 compared with the FMT HS group.

Fecal microbiota transplantation was used to investigate whether the modulation of the gut microbiota by XQLT could be involved in the favorable effect. The data showed that the FMT XQLT group and the FMT NS group had lower systolic blood pressures than the FMT HS group (Figure 8(a)). Fecal transfer from XQLT-treated rats more strongly affected systolic blood pressure than fecal transfer from other rats. The FMT XQLT group and the FMT HS group exhibited delayed body weight loss compared with the FMT HS group (Figure 8(b)). Cardiac function and organ weights were significantly improved after fecal transfer from XQLT-treated rats (Figures 9(a), 9(b), 9(c), 9(d), 9(e), 9(f), and 9(g)). Fecal microbiota transplantation from XQLT-treated rats contributed to more favorable effects on inflammation and fibrosis than transplantation from other rats (Figures 10 and 11). In addition, ZO-1 expression and intestinal mucosa injury were ameliorated after fecal transfer from rats in the XQLT group or the NS group compared with fecal transfer from rats in the HS group (Figures 12(a) and 12(b)). The level of serum endotoxin was significantly decreased after fecal transfer from XQLT-treated rats (Figure 12(c)).

### 3.8. XQLT Fecal Transplants Regulated the Composition of the Gut Microbiota and Increase the Levels of Butyrate

**Figure 13:**
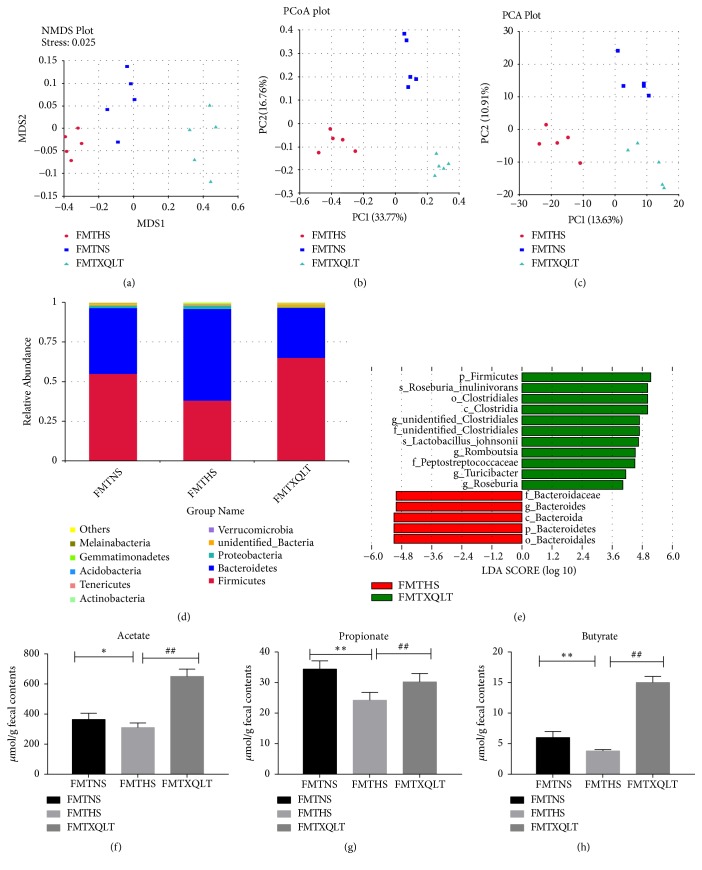
Analysis of the gut microbiota following transplantation from XQLT-treated rats. (a) Nonmetric multidimensional scaling (NMDS), (b) principal component analysis (PCA), and (c) principal coordinate analysis (PCoA) were performed between the NS group, the HS group, and the XQLT group. (d) Compositions of bacteria at the genus level. (e) LEfSe analysis of the bacterial taxa between the HS group and the XQLT group. (f, g, h) The levels of (f) acetate, (g) propionate, and (h) butyrate in feces. The data are presented as the mean±SD (n=5 rats per group). *∗* p < 0.05 and *∗∗* p < 0.01 compared with the FMT NS group; # p <0.05 and ## p <0.01 compared with the FMT HS group.

To confirm the effect of fecal microbiota transplantation on the composition of the gut microbiota, the composition of the gut microbiota was examined at the end of the study. Weighted PCoA, PCA, and NMDS analyses indicated that the composition of the gut microbiota was regulated by fecal microbiota transplantation (Figures 13(a), 13(b), and 13(c)). Furthermore, the FMT XQLT group and the FMT NS group had lower Firmicutes/Bacteroidetes ratios than the FMT HS group (Figure 13(d)). The abundance of Proteobacteria and Bacteroidetes was significantly increased in the FMT HS group compared to the FMT NS group but the FMT XQLT group performed a lower level of that. LEfSe analysis showed that the beneficial effects induced by fecal transfer from the XQLT-treated rats may be associated with changes at the genus level (Figure 13(e)).

The levels of acetate were not significantly different between the FMT HS group and the FMT NS group but were significantly increased in the FMT XQLT group (Figure 13(f)). The FMT NS group and the FMT XQLT group showed higher levels of propionate and butyrate than the FMT HS group (Figures 13(g) and 13(h)).

## 4. Discussion and Conclusion

It has been demonstrated that the progression of HFpEF is closely related to chronic inflammation. Recent evidence indicates that chronic inflammation is associated with dysfunction of the gut microbiota [13, 14]. However, previous evidence has shown that XQLT produces beneficial effects, such as anti-inflammatory effects, in the lung tissues of mice exposed to cigarette smoke concentrate (CSC) and lipopolysaccharide (LPS) [17] and increased gastrointestinal motility in the guinea pig ileum [18]. The effects of XQLT on HFpEF and the gut microbiota have not been investigated.

Our data suggested that XQLT treatment contributed to prevention of myocardial fibrosis, cardiac hypertrophy, and inflammatory cell infiltration in model rats. The XQLT group exhibited lower NT-proBNP levels and lower heart and lung weight ratios than the HS group. In addition, XQLT supplementation contributed to alleviate intestinal mucosal injury, altered the composition of the gut microbiota, and increased the levels of acetate, propionate, and butyrate. The results revealed that the treatment of XQLT contributed to the beneficial effects, such as the reductions in blood pressure, prevention of cardiomyocyte hypertrophy, and anti-inflammatory and antifibrotic effects, which were associated with altered gut microbiota. Whether the changes in the gut microbiota were essential for the favorable effects induced by XQLT cannot definitively be determined. These findings were supported by the results of fecal microbiota transplantation. Although antibiotics can disrupt the homeostasis of the intestinal microbiota, no evidence has shown that antibiotic treatment is beneficial for transplantation of gut microbiota [15]. Therefore, antibiotics were not used before transplantation of gut microbiota. As expected, our data suggested that the progression of HFpEF was effectively prevented by XQLT through dietary intervention or fecal transfer.

Previous evidence has indicated that chronic inflammation in model rats is associated with increased levels of endotoxin in the blood [23, 24]. The increases in the levels of endotoxin are triggered by gut dysbiosis and injury to the intestinal mucosa [25, 26]. Our data suggested that supplementation with XQLT contributed to reversal of the histology and tight junctions of the small intestine, reduced the levels of endotoxin, and downregulated the expression of MCP-1, TNF-*α*, and IL-6 in the model rats. To further understand the beneficial effects on the gut microbiota induced by XQLT, we analyzed the composition of the gut microbiota and the levels of short-chain fatty acids at the end of the experiment. Weighted PCoA, PCA, and NMDS analyses showed that supplementation with XQLT and fecal transfer from XQLT-treated rats changed the composition of the gut microbiota in model rats. At the phylum level, the ratio between Firmicutes and Bacteroidetes, which was used to measure gut dysbiosis, was decreased in model rats [27, 28]. The XQLT group and the FMT XQLT group performed a lower level of that. These results indicated that treatment with XQLT and fecal transfer from XQLT-treated rats contributed to reversal of gut dysbiosis in model rats. Previous evidence has indicated that butyrate has a beneficial effect on the histology and tight junctions of the small intestine [29, 30]. The XQLT group and the FMT XQLT group showed higher abundance of bacteria in the phylum Firmicutes than the HS group. Recent evidence indicates that special bacteria from the phylum Firmicutes promote the conversion of fiber to butyrate [31]. The data showed that the abundance of Lactobacillus was decreased in model rats, which could have increased the proportion of butyrate-producing bacterial strains. The XQLT group showed higher abundance of Lactobacillus than the HS group. As expected, the treatment with XQLT and fecal transfer from XQLT-treated rats conduced to the improvement of the histology and tight junctions of the small intestine. Furthermore, the beneficial effects observed in the XQLT group and the FMT XQLT group may have been associated with special alterations in the gut microbiota. Gram-negative bacteria promote inflammation by increasing the levels of endotoxin [32, 33]. The XQLT group and the FMT XQLT group exhibited lower proportions of gram-negative bacteria than the HS group and the FMT HS group. Administration of XQLT contributed to reduction of the abundance of Proteobacteria, which promotes cecal inflammation, systemic inflammation, and metabolic dysfunction [34, 35]. The improvements in intestinal morphology and the reductions in the proportions of gram-negative bacteria contributed to the lower levels of serum endotoxin. In addition, some evidence indicates that short-chain fatty acids can prevent chronic inflammation. LEfSe analysis showed that the beneficial effects of XQLT and of fecal transfer from XQLT-treated rats may be associated with changes at the genus level. The data showed that the levels of acetate, propionate, and butyrate were increased after treatment with XQLT and fecal transfer from XQLT-treated rats. These results are similar to those of previous studies [36], suggesting that XQLT may play a role like that of prebiotics to prevent the development of HFpEF in rats with hypertension.

The data indicated that treatment with XQLT prevented the progression of HFpEF via regulation of the composition of the gut microbiota, and our data seemed to exclude a direct influence on peripheral target issues. However, the changes in the gut microbiota caused by XQLT and the XQLT that was absorbed into the circulation may have both contributed to the prevention of the progression of HFpEF in the model rats. The favorable effects due to transplantation of gut microbiota may have been induced by metabolites of XQLT and/or metabolites produced by the gut microbiota that were altered by treatment with XQLT. Therefore, further studies will use the gut microbiota collected from the XQLT group to examine whether the beneficial changes were induced by the gut microbiota. In summary, this study provides strong evidence that the gut microbiota plays an important role in the progression of HFpEF. In addition, our results identify the mechanism of XQLT in preventing the progression of HFpEF and suggest a new method for the treatment of HFpEF.

## Data Availability

All data generated or analysed during this study are included in this published article.
